# Obsterical Society of London

**Published:** 1888-03

**Authors:** 


OBSTETRICAL SOCIETY OF LONDON.

*Specimens.*—Mr. MEREDITH showed two large pedunculated fibroid tumors, in both of which axial rotation had occurred, and in one to an extent involving occlusion of the cervical canal and retention of the menses.

Dr. LEWERS exhibited the cervix uteri, removed by supra-vaginal amputation on account of carcinoma, from a patient in whom abortion had been induced at the fourth month, a fortnight previously.

Dr. CARTER showed an epitheliomatus growth removed from the cervix by galvano-cautery.

Dr. W. DUNCAN exhibited ovaries and a piece of jejunum, the latter showing perforation after ovariotomy.

Dr. W. S. GRIFFITH showed a specimen of myxoma fibrosum of the chorion.

*On the Effect of Ergot on the Involution of the Uterus.*—A paper on this subject was read by Drs. G. E. HERMAN and C. O. FOWLER. They pointed out that the recommendation of a mixture containing ergot during the lying-in period was based upon a general knowledge of the action of such drugs and of the process of involution. No observations had been made, so far as they were aware, as to the actual effect of this treatment upon the process of involution. They had sought to ascertain its effect by measuring the height of the uterus above the pubes on successive days of the lying-in, in two sets of patients—one set (fifty-eight in number) treated with an ergot mixture for a fortnight after labor, the other set (sixty-eight in number) given a single dose of ergot after labor and no more. They found that in the cases treated by the continuous administration of ergot, the uterus diminished more rapidly in size than in those in which one dose only was given. They compared the two sets of cases as to the duration of the lochial discharge, but on this they did not find that the ergot treatment produced any appreciable effect.

Dr. BOXALL contrasted two series of cases, each referring to 100 patients. Every alternate patient admitted to hospital was given a mixture three times a day, containing ext. ergotæ amm. m. xv for a dose, during the first three days of lying-in. To avoid fallacy in the comparisons, the two series of observations were carried on simultaneously. The ergot mixture was given in the first series. In the second its routine administration was omitted, but in the series were included thirty-one patients for whom, on account of hæmorrhage, severe after-pains, etc., ergot was subsequently prescribed. The results were presented in a tabular form. By contrasting the two series of cases Dr. Boxall concluded: (1) That though the routine administration of ergot during the first three days of the puerperium exercised no appreciable effect on the date at which the lochia ceased (in this respect confirming the observations of the authors of the paper), the practice of giving ergot mixture during the three days following delivery tended to prevent the formation of clots and to hasten their expulsion and to diminish the frequency, intensity, and duration of after-pains. (2) That if omitted at first but given after, the ergot mixture tended to promote the expulsion of clots and to relieve after-pains. Dr. Boxall considered that (*a*) the routine practice which he had followed, of administering a douche at 110°–115° F., not only immediately after labor, but also twice a day during the puerperium until the lochia ceased (a powerful stimulant to the uterus); (*b*) the ergot which was given in every case immediately after labor, and (*c*) the ergot mixture which was prescribed subsequently in thirty-one of the cases included under the second series, all tended to lessen the difference which he had shown to exist between the two, and that in consequence the beneficial effect of the ergot mixture was even greater than that shown by the figures given in the tables.

Dr. DAKIN had made observations as to the effect of systematic administration of ergot for some days during the puerperium on cases in the General Lying-in Hospital while he was house physician. They did not support the view which the authors took, but showed that the average day when the fundus had sunk to the brim was 9.12 when ergot was given once only, and 10.3 when ergot was given daily for three days. There were, however, other fallacies than those named by the authors, for, in addition to the condition of bladder, rectum, thickness of abdominal wall, and weight of the uterus in the pelvis, there was the condition of the uterine axis, whether flexed or inclined anterio-posteriorly or laterally. He had found in a number of consecutive cases taken at random, that one-sixth of the uteri were in the axis of the body. These ought not to be compared with cases of anteflexion and version usual to the uterus during the puerperium, as there might be a difference of three inches or more on this account alone. In one of Dr. Dakin's cases the fundus was found one day in the left hypochondriac region nine inches from the pubes, whereas in the next it was to the right of the middle line, and only measured five inches and a half. He agreed with Dr. Boxall that the lochia were a better criterion of the rate of involution, and in this his own figures did not agree with the author's, for with one dose of ergot the average was 9.8, with three days of ergot 11.6. With reference to the retention of clots and the occurrence of after-pains, he found that, out of 92 cases where ergot was given for three days, 51 (55.4 per cent.) had after-pains, and 22 (23.9 per cent.) passed clots. Out of 103 cases where only one dose of ergot was given, 64 (62.136 per cent.) had after-pains, and 141 (3,592 per cent.) passed clots, so that the ergot cases had fewer after-pains but passed more clots. The unergotized cases, like Dr. Boxall's, passed clots up to the tenth day, whereas the ergotized ones passed no clots after the sixth day. It seems that the continuous use of ergot, by keeping up a tonic state of contraction, instead of allowing normal alternate contraction and relaxation, would tend to favor retention of clots and to prevent the normal process of involution. This was to a great extent borne out by his figures.

Dr. SWAYNE wished to know if chloroform was used during delivery, and in how many of the cases. In order to ascertain accurately the effect of ergot given after delivery, in his opinion it was necessary to remove all disturbing influences, such as the administration of anæsthetics during labor.

Dr. HERMAN, in reply, said that the cases observed by Dr. Dakin (which seemed to support an opposite conclusion to that arrived at by Dr. Fowler and himself) were only given ergot for three days, while their cases took it for a fortnight, and he did not therefore regard the two sets of cases as strictly comparable. The sources of error from the mode of measurement pointed out by Dr. Dakin had been present to the minds of Dr. Fowler and himself, but there was no other mode which was not attended with sources of fallacy. Such errors as arose from anteversion and anteflexion of the uterus were equally distributed among the two sets of cases, and so did not vitiate the comparison. Dr. Fowler and himself had paid particular attention to the occurence of lateral displacement, and had found that it depended, in the majority of cases, on the position in which the patient had been lying. They had not referred to it in this paper, as it did not seem to have any important bearing on the subject of the paper. In reply to the question of Dr. Swayne, chloroform had been given in six of the cases, namely, three of each series.

